# Development of forensic standards in China: a review

**DOI:** 10.1080/20961790.2021.1912877

**Published:** 2021-08-25

**Authors:** Xiaodan He, Chengtao Li

**Affiliations:** Shanghai Key Laboratory of Forensic Medicine, Key Laboratory of Forensic Science, Academy of Forensic Science, Ministry of Justice, Shanghai, China

**Keywords:** Forensic sciences, Standards, accreditation, technical committee, reform

## Abstract

Forensic science is crucial for the administration of justice and case investigation. In China, political-legal organizations, including the courts, public security, procuratorate, and judicial administration, developed their own forensic practices before 2004. As a result, the frequent and repeated appraisals undermined judicial authority and credibility. Thus, a law was published in 2005 to improve the uniform forensic management system by the Standing Committee of the National People’s Congress, leading to the establishment of the Forensic Administration of the Ministry of Justice in 2006. During this process, the increased accreditation and interflow highlighted the role of consensus in forensic standards for forensic service providers to avoid uncertainty regarding the methods used and interpretation of results. In 2017, a policy document was promulgated again to strengthen the importance of the uniform standards, which also proposed to establish a new national technical committee for the standardization of forensic science by the General Office of the State Council. In 2018, despite the continuing problems concerning uniformity, the Forensic Administration of the Ministry of Justice was merged into the Public Legal Services Administration. Yet, there is still a long way to go for the national technical committee for the standardization of forensic science. This paper analyses the evolution of forensic standards internationally and nationally, discusses the existing problems, and proposes relative solutions. Moreover, it discusses the future of standards development with the deepening of the reformation of both the national standardization and judicial system.

## Introduction

In modern lawsuits, forensic science covers all stages of criminal, civil, and administrative litigations. It provides technical support and professional services for law enforcement agencies to ascertain case facts, clarify liability attribution, and use the law correctly, and also provides relevant evidence by employing science and justice.

It is because of these important contributions of various forensic sciences to the reliability of legal outcomes that these applied disciplines and their possible pitfalls are necessary for a complete understanding and method verification before use. Without essential professional knowledge and a relevant support system, the forensic outcomes may be misused or misunderstood by case investigators. To minimize the risk of error, forensic science departments implement standard methods and processes to ensure comparability, consistency, and traceability of forensic outcomes.

Forensic standards have the following advantages: consistency of operations within a laboratory, consistency of operations among different laboratories and agencies, reliable and high-quality standards established for all forensic practitioners to make their work meet the specific forensic requirements, and judicial confidence in forensic outputs [1].

Forensic science standards not only guarantee consistency in laboratory practices and procedures, but also ensure the comparability and traceability of outcomes produced by individual laboratories, even among different countries. As a result, most countries in the world acknowledge the importance of developing and applying forensic standards.

## Evolution of international forensic standards

### International standard organizations and their standards

International standardization organizations (ISO) recognize the importance of standardization in forensic science, promoting the standardization process through technical committees and the development of relevant standards through the cooperation of different departments. The ISO, International Electrotechnical Commission (IEC) and International Telecommunication Union (ITU) are three international standardization organizations, among which ISO was the first to realize the standard’s importance for quality assurance of forensic laboratories. It published the ISO/IEC 17025 (General requirements for the competence of testing and calibration laboratories) (https://www.iso.org/standard/66912.html) and ISO/IEC 17020 (Conformity assessment—Requirements for the operation of various types of bodies performing inspections) (https://www.iso.org/standard/52994.html). The Standard ISO/IEC 17025 pertains to the organizational level and specifies laboratory mana­gement requirements, with an emphasis on policy and documentation. It does not address the requirements for sampling or testing at the crime scene. The Standard ISO/IEC 17020 provides criteria for inspection bodies in the examination of “materials, products, installations, plant, processes, work procedures or services” to provide certification.

Many countries, including Australia, the US, Canada, Columbia, and others, accredit their forensic science providers under the ISO/IEC 17025 standard. A few countries, such as Estonia, cover accreditation activities for forensic medical examinations of living persons and dead bodies under the ISO/IEC 17020. Additionally, the Netherlands has also introduced the ISO/IEC 17020 to crime scene activities and some other fields, like archaeo­logy [2].

The ISO Technical Committee of Forensic Science (ISO/TC 272) [3] was established in 2012. It has 28 participating and 17 observing country members as of 15 March, 2021. The secretary unit, set in Standards Australia (SA), is responsible for the standardization and technical guidance in the field of forensic science. The ISO/TC 272 now has two working groups (Review of terms and definitions, Annexes of ISO 21043-4) instead of five working groups that were disbanded in 2020, as well as one liaison organization (International Laboratory Accreditation Cooperation) and several liaison committees (ISO/CASCO, ISO/TC 292, ISO/TC 276, and others). The ISO/TC 272 has issued three international standards (ISO 21043-1:2018, ISO 21043-2:2018, and ISO 18385:2016), while three other international standards (ISO 21043-3, ISO 21043-4, and ISO 21043-5) are currently under development.

Additionally, there are many other ISO committees and standards relevant to forensic science. Joint ISO/IEC Technical Committee 1 (JTC1) [4] has three subcommittees that are developing standards including ISO/IEC 27042:2015, ISO/IEC 27037:2012, and ISO/IEC30121:2015 [5].

### National/regional organizations and their standards

#### America: ASTM, OSACs and ASB

In 1970, the American Society for Testing and Materials (ASTM) [6] Committee E30 on Forensic Sciences was formed. The Committee, with a current membership of over 600 people, has jurisdiction over 60 forensic standards. The standards still play a preeminent role in all aspects of forensic science, including criminalistics, digital and multimedia evidence, fire debris analysis, drug-testing analysis, collection and preservation of physical and digital evidence, as well as the reporting of findings.

The frequently quoted 2009 National Research Council (NRC) and National Academies of Sciences (NAS) report [7] on strengthening forensic science identified the lack of formal standards as a major issue. As a result, the Organization of Scientific Area Committees (OSACs) was founded in 2014 by the National Institute of Standards and Technology (NIST) and the Department of Justice (DOJ) to strengthen forensic science in the US [8]. The OSAC standards and documents include the OSAC Catalogs Document, Standards Developing Organization (SDO) standards, OSAC Discipline-Specific Baseline Documents, OSAC Technical Publications (coming soon), and OSAC Registry. The OSAC Catalog of External Standards and Guidelines compiled by the NIST is a collection of 700 standards, guidelines, and other documents applicable for OSAC members to assess existing standards and documents already publicly available [9]. It is important to note that the OSAC is not a government-recognized SDO. Therefore, to convert OSAC documents to formal standards, OSAC will need to partner with an SDO [10].

In 2015, the American Academy of Forensic Sciences (AAFS) standard Board (ASB) was established to provide accessible, high-quality, and science-based consensus forensic standards [11]. Different from the ASTM, the ASB is an American National Standards Institute-accredited SDO. The ASB does not directly compile standard documents, but endorses standards prepared by other organizations by specifying essential requirements for the standard formulation process [12].

### Australia: SA, SMANZFL and ANZPAA NIFS

As mentioned previously, Australia accredits its gover­nment laboratories against the ISO/IEC 17025. However, specific requirements, such as sample recognition and collection at a scene, appropriate sample packaging and labelling, transport of forensic samples, sample continuity, examination, interpretation of results, and reporting evidence, are not specifically covered by the 17025 standards. Thus, the Senior Managers of Australia New Zealand Forensic Laboratories (SMANZFL), working with the Australia New Zealand Policing Advisory Agency National Institute of Forensic Science (ANZPAA NIFS), deve­loped a framework for forensic *via* SA [13,14]. It is a novel approach and was the first time a holistic, non-discipline forensic standard has been developed that covers the whole period from crime scene to court. In four parts, this standard (AS5388) covers the recognition, recording, recovery, transport, and storage of material (Part 1), analysis of material (Part 2), interpretation (Part 3), and reporting (Part 4).

Additionally, SA issues some forensic technical standards, such as the Examination of Ignitable Liquids in Fire Debris (AS5239) [15], Procedures for Specimen Collection and the Detection and Quantitation of Drugs in Oral Fluid (AS4760), and minimizing the risk of contamination in products used to collect and analyse biological material for forensic DNA purposes (AS5483) [13]. The ANZPAA NIFS also plays an active role in technical standardi­zation, but only issues professional technical guidance.

### Europe: CEN/TC419 and ENFSI

The European Committee for Standardization (CEN) established the CEN/TC 419 Project Committee in 2012 to develop forensic standards. In 2017, TC 419 transferred all its work to ISO using the Vienna Agreement [16]. Now, TC 419 is developing the standards in collaboration with ISO [17].

The European Network of Forensic Science Institutes (ENFSI) develops forensic guidelines in Europe. Complying with the ISO 17025, ENFSI has been recognized as an expert association in forensic sciences, aiming to ensure the quality of forensic science throughout Europe. In addition, it publishes best practice manuals and guidelines. Currently, there are 15 best practice manuals and 14 forensic guidelines available for European countries [18].

## Evolution of forensic standards in China

### National organizations and their standards: SAC and AFS under the Ministry of Justice and TC179 under the Ministry of Public Security

Globally, national standards are generally developed by recognised national standards bodies. However, China National Standards are formulated by the government and issued by the China National Standardization Administration Committee (SAC). SAC is a competent organisation, authorized by the State Council of the People’s Republic of China to uniformly administer the national standardization operations [19].

In accordance with the Standardization Law provisions, the sector standards shall be developed by the relevant administrative departments under the State Council [20]. The responsibility of the Ministry of Justice for the uniform management of forensic science stems from the Decision on the Administration of Forensic Science (hereafter, the Decision), promulgated by the Standing Committee of the National People’s Congress in 2005. As a result, there was no competent department in charge of forensic standards before 2005, and the forensic standards used in practice can only refer to the standards developed by the rele­vant sectors, including the standards of criminali­stics, medical, and financial sectors for meeting the urgent needs of forensic activities. Among all relevant standards, the criminalistics standards are the most used in forensic science, accounting for about 39.8% [21].

The Technical Committee for the Standardization of Criminalistics (SAC/TC179) [22] was established in 1991, and its secretariat is the Institute of Forensic Science, Ministry of Public Security. The Committee is responsible for the research, development, and dissemination of standards in the field of crimina­l­istics. There are 502 standards in effect, including 40 national standards and 462 sector standards. The standards cover crime scene investigation, physical evidence extraction, inspection and identification, laboratory construction, and criminal technology products.

Considering the scope and content, the standards of forensic science and criminalistics do overlap, especially at the level of in-lab analysis. In recent years, with the development of public security technology and emphasis on standardization, TC179 has paid considerably more attention on in-lab analysis. From 2018 to 2019, it launched a special research programme on developing key forensic science standards [22]. Yet, their contents and emphases still differ. In China, the main task of technical institutions of public security departments is to discover, extract, and conduct laboratory analysis of physical evidence involving criminal cases with the purpose of providing clues for investigational activities. Therefore, they mainly focus on one-to-many retrieval analysis. Forensic expertise, administered by the Ministry of Justice, is used as evidence in court trials, convictions and sentencing, and focuses more on one-to-one identification. Therefore, the technological requirements are not completely consistent. As a result, forensic practitioners urgently need to develop technical standards that meet their functional orientation, developing direction, and technical requirements.

In fact, the earliest development of forensic technical standards in China can be traced back to the development of the “Standards for Evaluation of Serious Human Injury”, launched by the Institute of Forensic Science, Ministry of Justice (now the Academy of Forensic Science, hereafter AFS) in 1984. This standard is based on the metho­dology and technology of forensic medicine, providing a scientific basis and unified standard for the identification of serious injuries. This standard was promulgated jointly by the Ministry of Justice, the Supreme People’s Court, the Supreme People’s Procuratorate, the Ministry of Public Security, and Ministry of State Security on 29 March, 1990.

In 2001, the AFS explored the standardization of forensic techniques and issued the first batch of technical specifications for forensic toxicology and forensic biology. In 2003, it established a quality management system for laboratories/inspection bo­dies in accordance with the ISO/IEC 17025 and ISO/IEC 17020, also forming a few internal forensic methods, while being endorsed by the China National Accreditation Service (CNAS) [23]. In 2007, The SAC agreed to grant the judicial admini­stration standard code SF. Since 2010, the Ministry of Justice has organized institutions and experts with technical advantages and influence to develop, confirm, and promulgate 110 technical specifications for forensic expertise (SF/Z JD), which initially met the urgent needs of forensic activities, set up a preliminary framework for forensic science standards, and promoted their use [24]. In 2020, the Ministry of Justice further promulgated 20 forensic sector standards (SF/T) [25] in the form of ministry announcements and completed the filing at the SAC, keeping the sector standardization officially on track. Meanwhile, the AFS took the lead in becoming the first national forensic science service standardization demonstration unit in accordance with the series of national standards of *The Guidelines for Standardizations in Service Sector* and *Views on Advancing Pilot Project of the Service Standardization Guidelines* from the National Standards Committee. Additionally, the Standardization Administration of Shandong Province issued DB 37/T 3545 forensic standards, regulating the service standards from the aspects of service basis and service guarantee in 2019 [26].

At present, the forensic standards in use were mainly developed by the AFS under the Ministry of Justice and SAC/TC179 under the Ministry of Public Security, and they draw lessons from and refer to the technical standards of related sectors.

### Standards research in China

The study of forensic science standards in China has several forms [27–30]: (1) attention to and eva­luation of international standards, which are more concentrated in forensic medicine, such as the International Classification of Diseases by the World Health Organization, (2) research and evaluation of foreign advanced standards (such as Australia’s AS5388 standards, the ENFSI’s guidelines, and the Permanent Damaged Assessment Guideline of American Medical Association), (3) comparative studies of standards nationally and internationally (such as using the International Classification of Functioning, Disability and Health (ICF) for brain injury analysis), and (4) comparative study of domestic standards in different professional fields (such as between the occupational injury standards and injury classification standards).

In 2017, the *Opinions on Perfecting the Unified Administration System for Forensic Science* (hereafter, the Opinions) was issued by the government. The Opinions clearly put forward the unified management requirements of forensic science standardization. On the basis of this, some academic research findings regarding the construction of the standard law system, standardization management, and professional standard system have recently been published [31,32]. The theoretical research and systematic construction of forensic standardization have made progress in phases. Moreover, the Ministry of Science and Technology, the Ministry of Justice, and other ministries have established several projects about forensic standardization development. For example, among the seven sub-topics of the National Key Research and Development Project of the *Research and Application on the Demonstration of Forensic Science Innovation Technology*, two topics are focussed on the development of forensic science standardisations [33]: (1) the research on quality assurance of forensic competence and (2) the eva­luation system for forensic opinion evidence, such as capacity improvement, standard system, outcome judgement, standards development, and more.

### Standardization reform in China: association standard — a new form of standards

**Figure 1.**
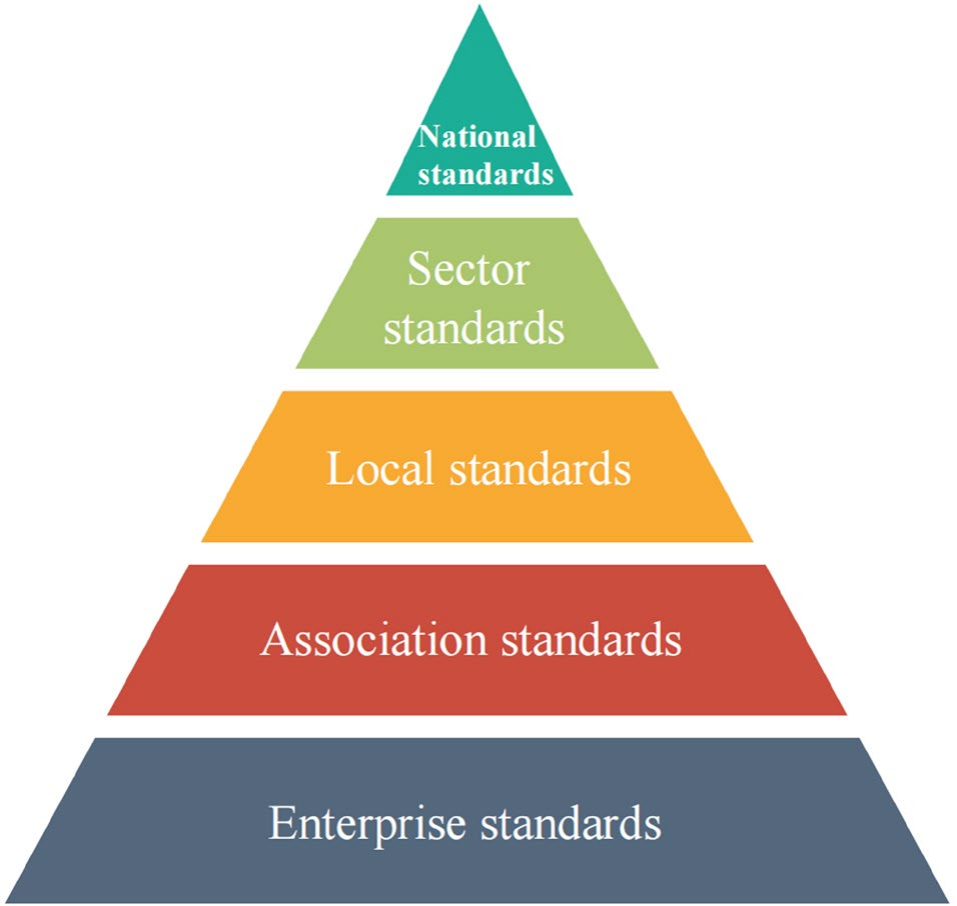
Chinese national standards system (five-level structure).

In 2015, the *Reform Plan for Deepening Standardization* was issued by the State Council, which was a landmark event in the history of standardization deve­lopment in China. It marked the beginning of a new stage of standardization reform and development. One of the most important standardization reform ideas was to transform the existing standard system from a single government supply pattern to a new pattern composed of government-led and market-determined standards. In January of 2018, the legal status of association standards was added and established following the implementation of new Standardization Law [20]. As a result, the Chinese national standards system changed from the former four-level structure to the current five-level structure (Figure 1).

The forensic science field responded rapidly, as forensic association standards have been emerging for 2 years. Through the national standardization information platform, an inquiry determined that forensic science association standards have cleared over 40 items and issued eight items by December 2020 [34]. The advantage of these standards is that the association members are flexible, representative, and extensive, and thus can carry on a thorough discussion. Additionally, the standards can be released quickly, allowing for rapid responses to address the constant technical progress of forensic science and needs of forensic practice.

## Challenges and solutions to forensic standards development

As mentioned above, China has made great efforts with the standardization and forensic system reform, mechanism innovation, and more. However, there are still many challenges involved with the standardi­zation of forensic science in China.

### Coordination and integration

Currently, the standards and methods used in forensic practice cover technical specifications, criminal sector standards, health care sector standards, financial sector standards, and more. The problems of dispersion and inconsistency are especially highlighted. In forensic clinical medicine, for example, the most influential standards are “Forensic Criteria of Human Injury Degree” and “Grading of Disability Degree of Human Injury”. However, they can only be released in joint document form issued by five ministries (the Supreme Court, Supreme Procuratorate, Ministry of Public Security, Ministry of Security, and Ministry of Justice) because of the inconsistent and uncoordinated understanding in practice. Meanwhile, there are some similar provisions in the *Forensic Assessment of Labour Ability: Grade of Injury and Disability of Occupational Diseases of Staff* (GB/T 16180-2014) issued by the Ministry of Human Resources and Social Security, *Personal Insurance Disability Assessment Criteria and Codes* (JR/T 0083-2013) issued by the Chinese Insurance Regulatory Commission, *Standards for the Evaluation of the Degree of the Employee’s Ability to Work without Disability or Illness* and *Medical Accident Classification Standard (Trial Implementation) of Ministry of Health*, as well as some local standards and guidelines. However, the concrete operation and execution are different, leading to conflict between standards. Thus, the same injured person could get different appraisal conclusions resulting from the application of different standards. To a certain extent, this can lead to the distrust of parties regarding these forensic opinions, the confusion of the judge when it comes to the adoption of the opinion, and damages to the judicial authority and credibility.

The key way to address this problem is to establish a unified technical management department of forensic science standardization that oversees the development, issuing, and interpretation of forensic standards. This principle was cleared in the Opinions described above. Now, the biggest difficulty is how to achieve coordination and integration between the Technical Committee for the Standardization of Forensic Science and the Technical Committee for the Standardization of Criminal Technology (TC179). Inter-ministerial joint meetings can be used to coordinate and communicate related standards.

### Standard system

With the rapid development of forensic science association standards, dispersion and inconsistency may be further aggravated. A Chinese national forensic science association does not exist as of this moment, and each province has its own forensic science association to develop the standards. As a result, the same case may easily have different forensic science outcomes in various provinces because of the application of different standards, which may lead to trouble in courts and affect judicial authority and credibility.

**Figure 2.**
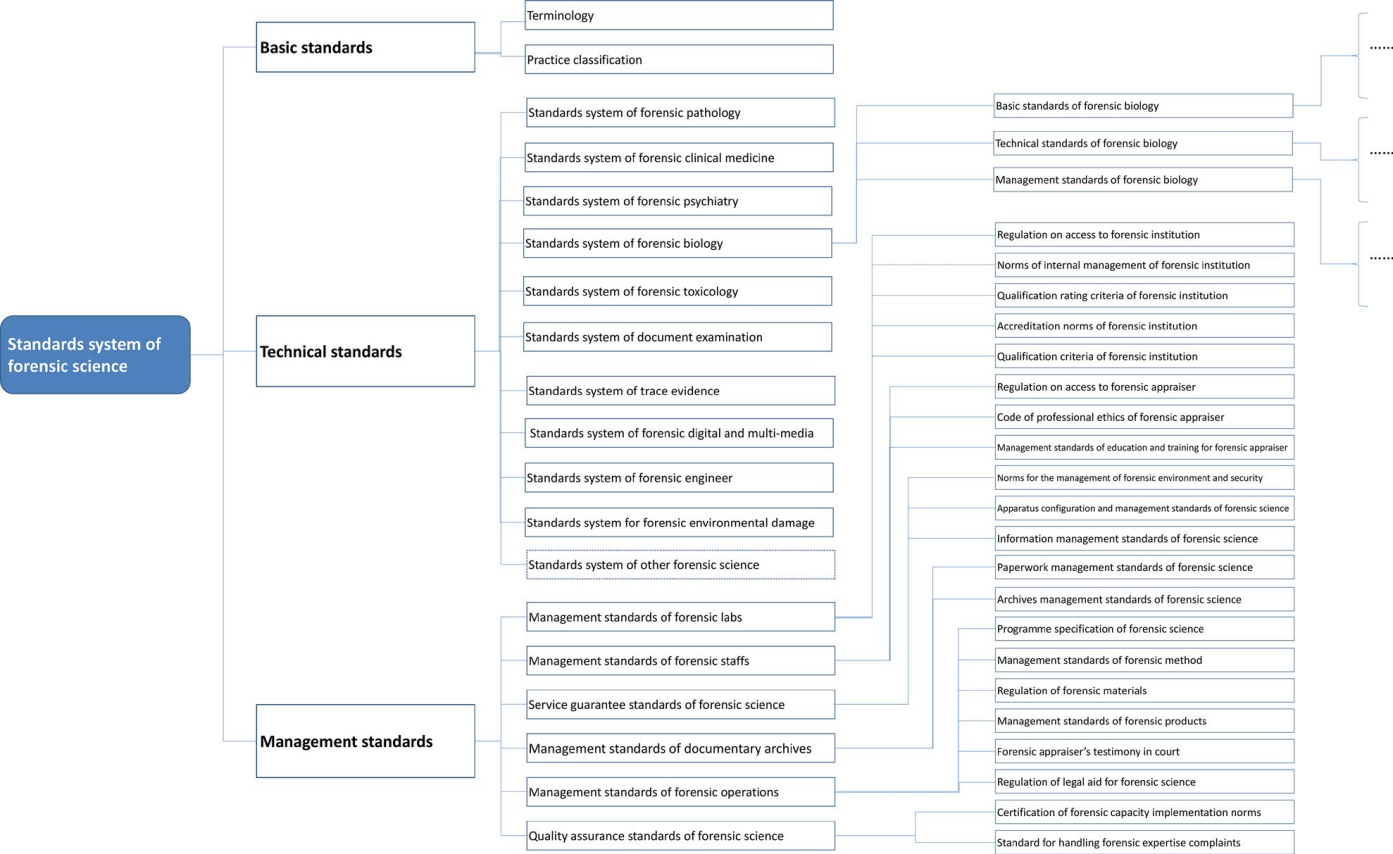
Standards system for forensic science issued by the Chinese Ministry of Justice in 2020.

**Figure 3.**
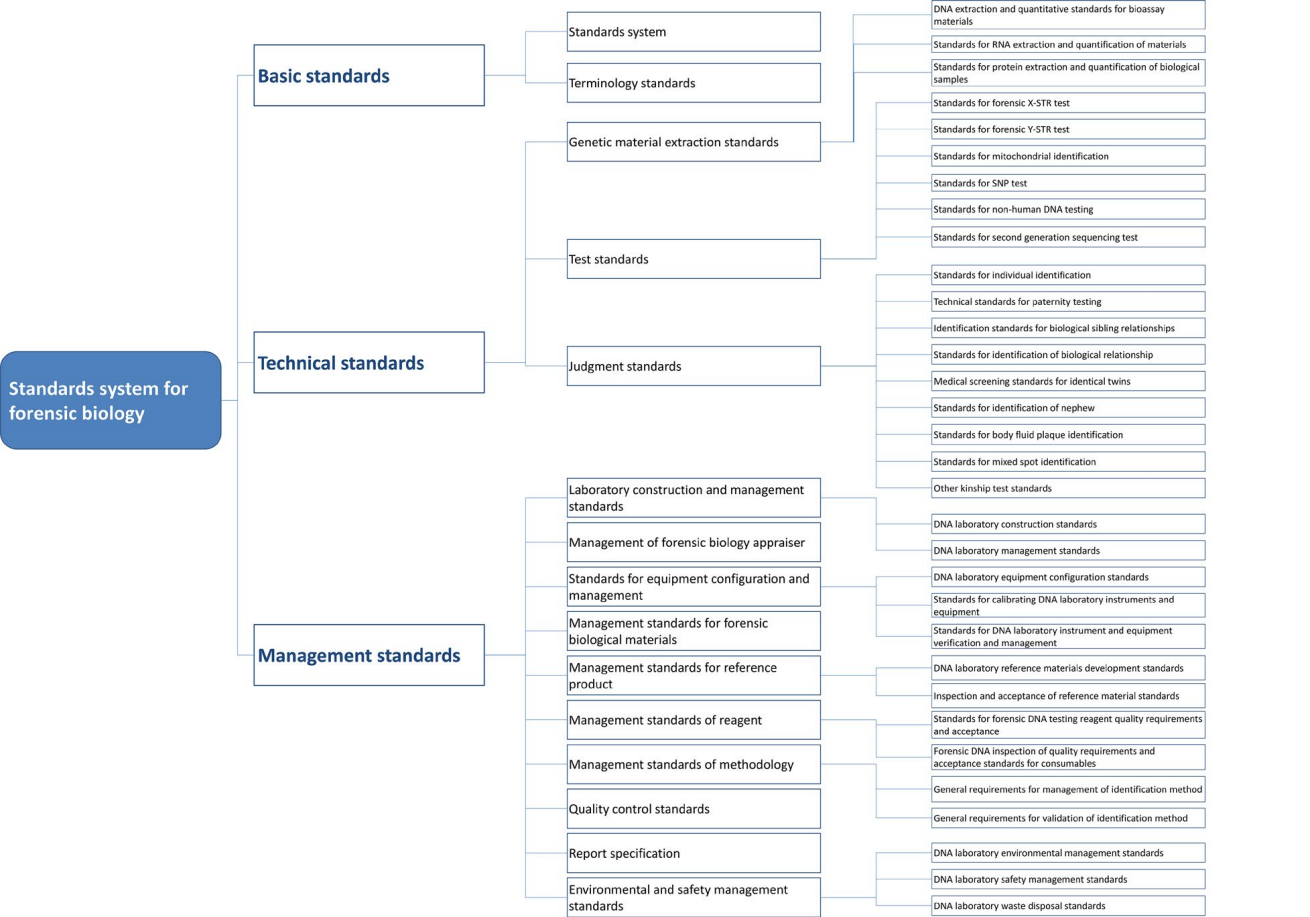
Standards system for forensic biology in China (adopted with permission [35]).

Strengthening the top-level design of forensic science standards is expected to solve the problem of dispersion and inconsistency. The goal is to construct a technically coordinated standard system with a reasonable structure and clear levels that successfully revises the standards and guarantees scientifically reliable results. By 2020, the Ministry of Justice issued a forensic standard system SF/T 0061-2020, as shown in Figure 2, in accordance with *the Principles and Requirements for Constructing Standard System* (GB/T 13016-2019). As an example, forensic biology identification shows the effective docking between the general standard system and the standard system of each specialty [35] (Figure 3).

The forensic system promotes the development of forensic science standards in the future. It can effectively eliminate the blind development of standards and ensure the consistency of standards from the start.

A potential problem is that the standard system table is only for guidance and reference (not mandatory), so it may not be followed in practice. Thus, dispersion and inconsistency of standards may likely still persist.

An additional problem is that TC179 also issues a standard system GA/Z1600-2019 [22]. We also compared these two systems. The structures of the two systems are both divided into three categories: basic standards, technical standards, and management standards. Among them, the basic standards and management standards are similar in composition. Basic standards cover terms, symbols, and classification standards, while management standards cover personnel, equipment, materials, methods, environment, records, and other quality control elements. This similarity also shows that ISO 17025 is widely used and accepted in judicial and public security forensic expertise.

The differences between the two systems are as follows:

First, the forensic standards under the framework of GA/Z 1600 are classified according to different forensic disciplines. Under the docking technical standard system, the standards are divided into crime scene investigation, forensic examination, forensic products, and others. The rapidly developing information technology standards are considered as a separate category, reflecting the technical needs of the police practice. Under the framework of SF/T 0061, the technical standards are divided scientifically by the 12 professional categories involved in forensic science. At the same time, they are connected with the standard system table of the next level (such as the standards system for forensic biology mentioned earlier) to reflect the needs and characteristics of each profession in the specific professional standard system, which is conducive to the innovation of specific professional technical standards.

Second, the composition of technical standards is different between the systems. For example, TC179 has a police dog technology working group that the Ministry of Justice does not have, while the Ministry of Justice has relevant forensic evaluation on environmental damage that the TC179 does not have. This results in different standard groups between GA and SF standards.

Third, the determination methods for the standardization objects in every standard document are different, including for toxicological analysis. TC179 develops a technical standard with a target object, while the Ministry of Justice develops a technical standard with a target for a class of objects.

The coexistence of the two standards systems may inevitably produce problems of dispersion and inconsistency.

### Accreditation and certification

The accreditation activities of forensic science in China began with the 2005 Decision issued by the NPC Standing Committee. In 2012, a joint notice was sent by the Ministry of Justice and the Certification and Accreditation Administration, requesting forensic institutions throughout the country to participate in certification and accreditation. From this moment, certification and accreditation have become powerful starting points and a regular working mode for the supervision of forensic science and improvement of the institutions’ own capacity. As of January 2019, a total of 546 institutions in China have passed laboratory accreditation [36].

In the existing documents of quality assurance of China, Article 4.5.14 of *the General Requirements for Qualification Evaluation of Inspection and Testing Institutions*, Article 4.5.12 of *the Evaluation of Competency in Qualification Recognition of Inspection and Testing Institutions and Forensic Science Institutions*, Article 4.1.1 of the *Guidelines for Quality Assurance of Forensic Process*, and Article 7.2 of *the Guidelines for Accreditation of Forensic Science Institutions* specify that the accreditation body shall establish and maintain the method control procedures and stipulate the principles and procedures for the selection, verification, and validation of methods.

The method control programme is in accordance with the requirements by observing whether the forensic institutions can accurately choose the suita­ble standards, verify the proper standards, and validate the non-standard method in accreditation activities.

As one of the outputs of certification and accredi­tation activities, method evaluation helps the institutions with standard selection and application, as well as adopts various ways, such as training and education, to achieve supervision, inspection, and continuous method improvement.

Strengthening the coordination through accreditation activities can effectively promote the implementation of standards in forensic activities and deeply ensure the continuous improvement of the forensic community.

### Technical innovation

Standards form a solid platform that carries scientific and technological innovation. The authority and scientific nature of technical standards can promote the popularization and application of technological innovation achievements. Through the “common use and reuse” after the standard of “consensus”, the new technology will play a major role in determining problems in practice, help address these problems, and establish a “popularized and replicable” realistic path for development and change. Undoubtedly, scientific and technological innovation can effectively promote the development of standards.

However, the transformation of the achievements of new technologies requires careful treatment. The standards must be derived from empirical proof of the validation and accuracy of the method. During the 12th Five-Year Plan and the 13th Five-Year Plan period, China has overcome a few major technical problems with forensic science and developed a series of key technical standards. However, some standards are not frequently adhered to. The forensic standards implemented by forensic institutions are reportedly less than a quarter of the total number of existing standards [37].

A major challenge in the future will be to verify the methods formed by scientific and technological innovation, determine the validation, reliability, and accuracy of the methods, and achieve a balanced development of standard innovation and populari­zation. SAC/TC 179 developed both GA/T1649-2019, “Specifications for validation of examination methods for toxicants”, and GA/T 1674-2019, “Specifications for validation of morphological comparative methods for trace examination” [22].

### Internationalization

China has a relatively sound international standardization working system, but it participated in the ISO/TC 272 forensic work relatively late. In 2016, AFS sent members to the Working Group of ISO/TC 272 for the first time. However, China was an observing member of ISO/TC 272 before 2020, but now is a participating member. The transition from Member O to Member P marks an important step forward in the internationalisation of forensic science standards in China, from spectator and learner to participant. The latter parts of ISO 21043, particularly the application of statistics to the interpretation of forensic evidence, have received extensive attention from Chinese scholars [38,39]. In the future, the standardization of forensic science in China will continue exploring the development of standards based on quality management, putting forward proposals for universal international standards, and striving to transform from a participant to a contributor and promoter.

## Conclusion

The *Opinions on Accelerating the Construction of Public Legal Service System* issued by the General Office of the State Council in 2019 emphasizes an improvement of the unified forensic science ma­nagement system, strengthens the link between forensic science management and judicial case handling, unifies the forensic science standards, and provides technical support for case fact determination. The *Opinions on Further Deepening of the Reform and Enhancing the Credibility of Forensic Science* was also published in 2021.

With the deepening of standardization and judicial systems reform, as well as the development of forensic science in China, the development of China’s forensic science standards will likely continuously improve the management system and operating mechanisms, carry out research and development of key technical standards, connect with certification and accreditation activities, and form a good circular mechanism for the continuous improvement of the standard system. In addition, it will begin the process of continuous construction and development of forensic science standardization in China to establish the most high-quality and healthy forensic science field possible.
